# The Transcription Factor SsSR Mediates Ergosterol Biosynthesis and Virulence in *Sclerotinia sclerotiorum*

**DOI:** 10.3390/jof11070509

**Published:** 2025-07-05

**Authors:** Huihui Zhao, Xiaofan Liu, Jintao Jiang, Jiatao Xie, Yanping Fu, Yang Lin, Tao Chen, Bo Li, Xiao Yu, Xueqiong Xiao, Xueliang Lyu, Weidong Chen, Daohong Jiang, Jiasen Cheng

**Affiliations:** 1 National Key Laboratory of Agricultural Microbiology, Huazhong Agricultural University, Wuhan 430070, Chinajiataoxie@mail.hzau.edu.cn (J.X.); yanglin@mail.hzau.edu.cn (Y.L.); taochen@mail.hzau.edu.cn (T.C.);; 2 The Provincial Key Lab of Plant Pathology of Hubei Province, College of Plant Science and Technology, Huazhong Agricultural University, Wuhan 430070, China; 3 United States Department of Agriculture, Agricultural Research Service, Washington State University, Pullman, WA 99164, USA

**Keywords:** *Sclerotinia sclerotiorum*, transcription factor, virulence, ergosterol, stress tolerance

## Abstract

*Sclerotinia sclerotiorum*, known as a typical necrotrophic pathogenic fungus, exhibits a complex pathogenic mechanism. Research on *S. sclerotiorum* has primarily focused on oxalic acid, pathogenicity-related enzymes, and secreted proteins. In this study, we identified a transcription factor, SsSR (*S. sclerotiorum* Sterol-Related transcription factor), which regulates *S. sclerotiorum* infection by modulating virulence through ergosterol biosynthesis. We characterized the transcriptional activity of *SsSR* and its downstream target gene, *SsCYP51*. *SsSR* undergoes phosphorylation induced by the host plant, subsequently regulating the expression of *SsCYP51*. The deletion of *SsSR* or *SsCYP51* does not affect the growth or acid production of *S. sclerotiorum*, but it leads to a reduction in ergosterol, significantly diminishing virulence and impairing the stress tolerance of the hyphae. In summary, this study identifies a transcription factor, *SsSR*, that specifically regulates the virulence of *S. sclerotiorum*. *SsSR* upregulates the expression of *SsCYP51* through phosphorylation during the infection phase, leading to the synthesis of ergosterol, which enhances hyphal stress tolerance and thereby promotes infection.

## 1. Introduction

*Sclerotinia sclerotiorum* is a typical necrotrophic fungus with a broad host range capable of infecting over 700 plant species, leading to significant economic losses [1,2]. Among the crops affected, sclerotinia stem rot in oilseed rape is particularly damaging to agricultural production, and its prevention and control remain challenging. Understanding the pathogenic mechanisms of *S. sclerotiorum* is therefore crucial for developing effective management strategies [3]. Current research has focused on OA (oxalic acid), cell-wall degrading enzymes, and secreted proteins. To establish infection, *S. sclerotiorum* employs several strategies. First, it produces cell wall-degrading enzymes to break down cellulose and hemicellulose in the plant cell wall [4]. Second, it synthesizes oxalic acid, which creates an acidic environment conducive to infection by killing plant cells, scavenging oxidative burst, and inhibiting autophagy [5,6,7,8,9]. Additionally, secreted proteins also play essential roles in the virulence. For example, *S. sclerotiorum* can secrete effector proteins such as SsPEIE1, SsCP1, SsCVNH, and SsPINE1, which suppress the host’s immune response [10,11,12,13].

Gene regulation by transcription factors (TFs) plays an essential role in the virulence of phytopathogens. Understanding these regulatory networks is crucial for identifying novel targets for disease control. TFs typically belong to families such as the zinc finger structure, bZIP family, and HD/Hox families [14]. In plant pathogenic fungi, the role of TFs in virulence has been extensively studied, particularly in *Fusarium graminearum* and *Magnaporthe oryzae*. For example, the transcription factor FgPacC suppresses the transcription of iron uptake genes, and the bZIP transcription factor FgHAPX regulates the iron storage gene, mitigating the adverse effects of high iron concentrations on the mycelium in *F. graminearum* [15,16,17,18]. The production of the DON toxin, a critical factor in *F. graminearum* virulence, is regulated by APSES-type TFs. FgStu interacts with the acetyltransferase SAGA to regulate *TRI* gene expression, thereby enhancing DON production during infection [19]. Similarly, in *M. oryzae*, key virulence processes such as conidiogenesis, appressorium formation, and infectious growth are tightly regulated by TFs [20]. The histone methyltransferase MoSET1 works in conjunction with the transcription factor MGG_06898 to regulate spore production, while Vrf1 and Hox7 influence appressorium formation [21,22]. MST12, as a zinc finger transcription factor downstream of the protein kinase PMK1, plays a key role in the growth of invasive mycelium [23]. These studies highlight the critical role of TFs in regulating the virulence of phytopathogenic fungi, and elucidating their functions could reveal specific targets for effective disease control strategies.

TFs also play a critical role in the growth and virulence of *S. sclerotiorum*. The SsSnf5-SsHsf1 transcriptional module has been found to regulate growth, virulence, and oxidative stress response by controlling the expression of *HSP* genes [24]. Additionally, SsMads and Sssfh are involved in growth and virulence regulation, while the atypical forkhead (FKH)-box family transcription factor SsFkh influences not only growth and virulence but also sclerotia development [25,26,27]. While these TFs significantly regulate the growth of *S. sclerotiorum*, the reduction in virulence may involve more complex mechanisms, with a substantial contribution likely linked to the decreased growth rate. In summary, most of these limited studies on *S. sclerotiorum* TFs are on growth regulation. Notably, no TFs have been reported to specifically regulate virulence in *S. sclerotiorum*.

In this study, we identified a zinc finger-containing domain in the transcription factor SsSR of *S. sclerotiorum*. Deletion of *SsSR* did not affect growth but significantly reduced virulence and stress tolerance, including osmotic stress and oxidative stress. SsSR plays a critical role in regulating the expression of *SsCYP51* and other genes involved in fungal ergosterol biosynthesis. *SsSR* mutants exhibited defects in ergosterol biosynthesis, leading to compromised plasma membrane integrity. *SsCYP51* is also essential for virulence and stress tolerance, similar to the effects observed in *SsSR* mutants. Notably, overexpression of *SsCYP51* in the Δ*SsSR* background rescued the Δ*SsSR* phenotype, providing genetic evidence that both *SsSR* and its downstream gene, *SsCYP51*, are crucial for the full virulence of *S. sclerotiorum*.

## 2. Experimental Procedures

### 2.1. Strain Culture and Plant Culture Conditions

The *S. sclerotiorum* wildtype strain 1980 (ATCC 18683) was cultured on PDA (200 g potato, 20 g glucose, 10 g agar per L); the knockout mutants Δ*SsSR*-3, Δ*SsSR*-4, and Δ*SsCYP51* were cultured on PDA containing 50 µg/mL hygromycin B (Biofroxx, 1366ML010); and the complementary transformants Δ*SsSR*-C-5 and Δ*SsSR*-C-6 were grown on PDA containing 200 µg/mL G418 (Yeasen, 60220ES08, Shanghai, China). All these strains were cultured at 20 °C and stored on PDA at 4 °C.

The *B. napus* and *A. thaliana* plants were grown at 22 °C (12 h light/12 h dark cycle) in a greenhouse.

### 2.2. Gene Deletion and Genetic Complementation

Gene deletion mutants of *SsSR* and *SsCYP51* were obtained using the homologous recombination and split-tagging method [28]. The knockout strategy is illustrated in Supplementary Figure S1A. Two fragments of about 1000 bp each, *SsSR*/*SsCYP51*–5′ and *SsSR*/*SsCYP51*–3′, flanking the gene ORF were amplified from genomic DNA by PCR with primers *SsSR*/*SsCYP51*-UP-PUCH18-F/R (both containing SalI sites) and *SsSR*/*SsCYP51*-DOWN-PUCH18-F/R (both containing XbaI sites). These PCR products were ligated into SalI-digested PUCH18 and XbaI-digested PUCH18, respectively. The upstream transform-fragments *SsSR*/*SsCYP51*-Up-HY were amplified using primers *SsSR*/*SsCYP51*-UP-F, HY-R and the downstream transform-fragments *SsSR*/*SsCYP51*-YG-Down were amplified using primers *SsSR*/*SsCYP51*-Down-F, and YG-R were transfected into protoplast of the wildtype strain 1980 using PEG-mediated protoplast transformation to obtain the *SsSR*/*SsCYP51* knockout transformants [29]. Deletion mutants were identified by PCR.

Complementary strains were also obtained using protoplast transformation. *SsSR/SsCYP51* full-length fragment and *SsSR* fragments with phosphorylation site mutations were ligated into XhoI/KpnI-digested PCETNSF. Then, we transferred the promoter-*SsSR*-flag-PtrpC-NptII-TtrpC fragments into *SsSR* mutant strain protoplast to obtain complementary strains. The complementary strains were verified by Western blot. All the primers used to amplify are listed in Table S1.

### 2.3. Pathogenicity Assays

For detecting the fungal pathogenicity, agar discs (2/3 mm in diameter) were punched from 2 × SY (0.5% (*w*/*v*) sucrose and yeast extract, 1% (*w*/*v*) agar and inoculated onto the leaves of 4–5-week-old Arabidopsis plants or 6–7-week-old rapeseed, which were then incubated at 20 °C. Photographs were taken, and necrotic lesions were measured 24/36 h post-inoculation. In each group of Arabidopsis or rapeseed leaves, after measuring the lesion area, equal area samples were taken from the infected sites using a 1.5/2.5 cm diameter punch. The DNA of the samples was extracted and analyzed for the relative content of fungal pathogens and plant DNA by qPCR.

### 2.4. Analysis of Growth, Acid Production, and Mycelial Tip Morphology

The strains were inoculated on PDA for three generations and then transferred to 17.5 mL PDA. The radius of mycelial colony was measured at 36 h and 48 h, and the growth rate of *S. sclerotiorum* was recorded and analyzed; the strains were inoculated on quantitative 17.5 mL PDA containing bromophenol blue indicator (0.005%), and the oxalic acid production of *S. sclerotiorum* was observed after 36 h and photographed. The strains were inoculated on 17.5 mL PDA for 36 h, and the morphology of the mycelial tips of *S. sclerotiorum* was observed and photographed under an optical microscope (VHX-6000, Keyence, Japan).

### 2.5. Stress Response Assays

To test the sensitivity under different stress conditions, mycelial plugs (5 × 5 mm) of the wildtype strain 1980 and the mutants were inoculated in the center of PDA with various stressors, including osmotic stress by adding 1 M NaCl and oxidative stress by adding 10 mM H_2_O_2_.

### 2.6. RNA Extraction and RT-qPCR

After the collected different samples were ground into powder with liquid nitrogen, RNA was extracted using 1 mL of Trizol. The EasyScript^®^ One-Step gDNA Removal and cDNA Synthesis SuperMix kit was used for genomic DNA digestion and cDNA synthesis (TransGen Biotech, Beijing, China). The RT-qPCR assays were performed using a Bio-Rad CFX96 instrument (Bio-Rad, Hercules, CA, USA) or a CFX384 instrument (Bio-Rad, Hercules, CA, USA). The procedure used was an initial 95 °C denaturation step for 5 min followed by 40 cycles of denaturation for 30 s at 95 °C, annealing for 30 s at 60 °C, and extension for 30 s at 72 °C. The *S. sclerotiorum* β-tubulin gene *Sstublin* (*SS1G_04652*) was used as reference to normalize the relative expression levels [30], and the method of 2^−ΔΔCt^ was used for analysis [31]. Sequences of primers used in the RT-qPCR assays are listed in Table S2.

### 2.7. ChIP(Chromatin Immunoprecipitation)-qPCR

ChIP was performed according to two described protocols with additional modifications [32,33]. The samples that were cultured on the PDA for 2 days were fixed and crosslinked with 1% formaldehyde for 15 min and then treated with 125 mM glycine for 5 min to terminate the crosslinking. The samples were washed twice with 1 × PBS, ground into powder with liquid nitrogen, and lysed by adding 1 mL of RIPA strong lysis solution (Beyotime, Shanghai, China) for 1–2 h. The samples were centrifuged at 4 °C and at 12,000 rpm for 10 min; then, the supernatant was obtained and broken by ultrasonication (ultrasonication for 10 s, resting for 10 s, 20 cycles). After centrifugation, 100 µL of supernatant was removed, and 900 µL of freshly prepared ChIP solution (16.7 mM Tris-HCl, pH = 8.0, 1.1% TritonX-100, 1.2 mM EDTA, 167 mM NaCl) with anti-Flag beads was added for overnight incubation at 4 °C. On the next day, samples were washed once with low salt, once with high salt, once with LiCl, and twice with TE buffer (pH = 8.0), and for each wash the samples were incubated on a shaker at 4 °C for 10 min followed by 4000 rpm for 5 min. Elution could be performed at room temperature. The sample with 200 µL of a freshly formulated ChIP elution solution (1% SDS, 0.1 M NaHCO_3_) was incubated at room temperature for 10 min andcentrifuged at 12,000 rpm for 1 min, then the supernatant transferred to a new EP tube. The elution step is repeated 3 times to obtain 600 µL of eluate. To the 600 µL elution solution, 48 µL of 2.5 mM NaCl was added, and 50 µL input was added to 350 µL of elution solution and 32 µL NaCl, and treated at 65 °C for 8 h to remove formaldehyde crosslinks.

After de-crosslinking, the samples were treated with proteinase K at 37 °C for 2 h. For DNA purification, 300 µL of DNA-extracting phenol and 300 µL of chloroform were added to the samples and centrifuged. The supernatant was treated with twice the volume of anhydrous ethanol at −80 °C for 1 h, centrifuged to remove the supernatant, and the precipitate was washed once with 75% ethanol and dissolved by adding 40 µL TE buffer.

The obtained ChIP-DNA was analyzed by qPCR according the protocol [34] using the procedure of 95 °C denaturation step for 10 min followed by denaturation for 15 s at 95 °C and annealing for 30 s at 60 °C for 39 cycles.

### 2.8. Yeast One-Hybrid (Y1H) Assays

The CGAA-containing motif on the *SsCYP51* promoter was ligated into the pAbAi. The p*SsCYP51*-pAbAi was digested with Bstb1 at 65 °C for 1 h and then co-transformed with PGADT7 or SsSR-PGADT7 to Y1H according to the yeast transformation kit (Weidibio, Shanghai, China). After incubation at 30 °C for 3 days, a single colony was selected for PCR verification, and the successfully co-transformed yeast was delineated. Transformants were diluted to a concentration of OD = 0.002 and transferred to SD-Ura/Leu (Coolaber, Beijing, China) and SD-Ura/Leu supplemented with ABA.

### 2.9. Ergosterol Extraction and High-Performance Liquid Chromatography Analysis

Ergosterol extraction methods were mainly based on the described protocol [33]. After various strains were cultured on cellophane-lined PDA for 2 days, the mycelium was collected and dried at 60 °C and then ground. Next, 0.1 g of *S. sclerotiorum* dried powder was saponified in 10 mL of a mixture of methanol–chloroform (3:1) at room temperature overnight, and then added in turn to 10 mL of water, 10 mL of chloroform, and 10 mL of a 0.5 M phosphate buffer containing 2.0 M KCl in 0.5 M phosphate buffer. After layering, the extracted chloroform phase was blown dry at 60 °C on a nitrogen blower instrument (Nitrogen, Auburn, CA, USA). After blow-drying, we added 10 mL of methanol and ethanol (4:1) mixture containing 1.4 M KOH and processed it at 60 °C for 1 h, added 10 mL of petroleum ether (boiling range 60–90 °C), took the petroleum ether layer and blow-dried it on a nitrogen blower instrument, added ethanol to dissolve it, and then filtered it with a microporous membrane filter (Φ 0.45 µm). The filtered sample was placed at 4 °C for later use.

HPLC analysis of ergosterol in *S. sclerotiorum*: The chromatographic column was Agilent TC-C18 (5 µm, 4.6 × 250 mm) (Agilent Technologies, Santa Clara, CA, USA), the mobile phase was 100% methanol, the column temperature was room temperature, the flow rate was 1 mL/min, the detection wavelength was 282 nm, the injection volume was 50 µL, and the peak appearance time was about 5 min. The ergosterol standard was dissolved in anhydrous ethanol and diluted to 320, 160, 80, 40, 20, 10, 5, 2.5 mg/L. The linear regression equation was made according to the peak time and peak area corresponding to the different concentrations of the standard, and the ergosterol content in the sample was calculated based on the peak area of the sample.

### 2.10. Measurement of Membrane Permeability

For PI staining, the method used was according to the protocol [35]. Protoplasts of strains were obtained by lysing *S. sclerotiorum* mycelia for 1 h at 30 °C using 0.7 M NaCl configured with 1% lytic enzyme followed by filtration with triple rubbing paper, and then, centrifugation and resuspension with 1 mL of STC. The prepared protoplasts were stained with 10 µL of 1.5 mM PI for 15 min in the dark, centrifuged at 400× *g* for 5 min, resuspended in STC, and analyzed using flow cytometer (cytoflex-LX, Beckman (Brea, CA, USA)).

### 2.11. Statistical Analysis

The statistical significance of data was determined by one-way analysis of variance (ANOVA) or two-way ANOVA, and graphs were generated by Prism 8 (GraphPad Software, Boston, MA, USA). Data presented are the mean ± SD. The significant difference was evaluated at a *p* of <0.05.

## 3. Results

### 3.1. The Expression of the Transcription Factor SsSR Was Significantly Upregulated During Infection

**Figure 1:**
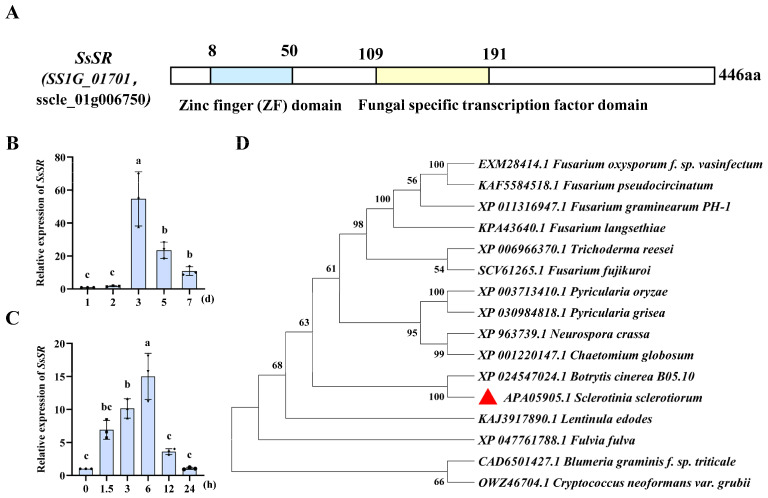
SsSR is a fungal-specific transcription factor that is induced by the host. (**A**) Conserved domains of SsSR identified using the NBCI protein database. (**B**) Relative levels of transcript accumulation of *SsSR* were determined by RT-qPCR when cultivated on PDA at 20 °C for 1–7 days. The relative levels of transcripts were calculated using the comparative Ct method. The levels of *β-tubulin* transcript of *Sclerotinia sclerotiorum* were used to normalize different samples and the wildtype strain 1980 inoculated for 1 day was used as a control. Values are the means of three independent trials. Different lowercase letter (a, b, c) represented significant differences between groups (*p* < 0.05). (**C**) Relative levels of transcript accumulation of *SsSR* were determined by RT-qPCR when inoculated on rapeseed leaves at 20 °C for 0–24 h. The relative levels of transcripts were calculated using the comparative Ct method. The levels of *β-tubulin* transcript of *S. sclerotiorum* were used to normalize different samples, and the wildtype strain 1980, inoculated for 0 h, was used as a control. Different lowercase letter (a, b, c) represented significant differences between groups (*p* < 0.05). (**D**) Phylogenetic analysis of SsSR. Branch length is proportional to the average probability of the change in features on that branch. Phylogenies were constructed using Mega 11 with the maximum-likelihood algorithm. *S. sclerotiorum* sequence (APA14016.1) was marked with red triangle.

Analysis of RNA sequencing (RNA-Seq) data from the infection stage of *S. sclerotiorum* revealed a significant up-regulation in the expression level of *SsSR* (*SS1G_01701*, *sscle_01g006750*) [36]. NCBI domain analysis indicated that SsSR is a 446 amino acid transcription factor possessing a conserved zinc finger domain and a fungal-specific transcription factor domain (Figure 1A). To define the regulation of *SsSR* expression more precisely, we examined its transcript accumulation across developmental and infection stages by quantitative reverse transcription–polymerase chain reaction (qRT-PCR). The results demonstrated that *SsSR* was transcriptionally up-regulated 3 days after inoculation on PDA and 6 h post-inoculation on the plant (Figure 1B,C), consistent with previous transcriptome results. In addition, phylogenetic analysis indicated that SsSR is orthologous to the transcription factor FgSR (sterol uptake control) (Figure 1D). These results suggest that *SsSR* may play an important role in growth and virulence.

### 3.2. SsSR Is Essential for the Full Virulence of S. sclerotiorum

**Figure 2:**
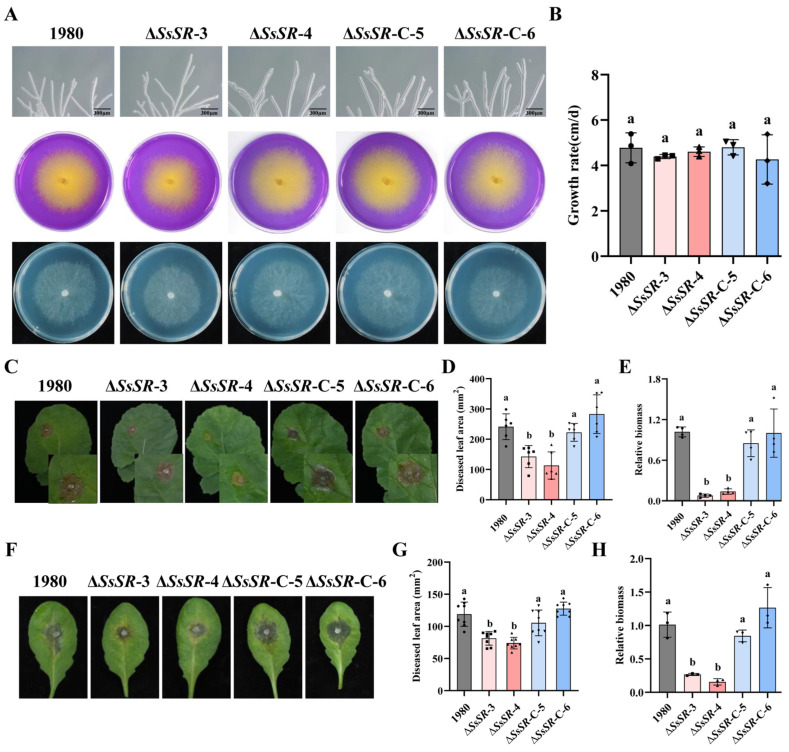
SsSR is essential for the full virulence of *S. sclerotium*. (**A**) Mycelial tip morphology, oxalic acid production, and colony morphology of the wildtype strain 1980, Δ*SsSR*-3, Δ*SsSR*-4, Δ*SsSR*-C-5, and Δ*SsSR*-C-6 strains on PDA at 20 °C. Photographs were taken 36 h after inoculation. (**B**) Growth rates of above strains on PDA at 20 °C. (**C**,**F**) Disease symptoms on the detached *B. napus* and *Arabidopsis* leaves inoculated with *Sclerotinia sclerotiorum* wildtype strain 1980, *SsSR* mutants (Δ*SsSR*-3, Δ*SsSR*-4), and complementary strains (Δ*SsSR*-C-5 and Δ*SsSR*-C-6). Photographs were taken at 36 h post-inoculation. (**D**,**G**) Lesion areas were measured by the cross-over method. (**E**,**H**) DNA was extracted from tissue samples of mycelium and leaves at lesion sites for detecting the number of pathogen and plant endogenous genes using RT-qPCR. Then, the CT values obtained were used to analyze relative biomass. Different lowercase letter (a, b) represented significant differences between groups (*p* < 0.05).

To evaluate the biological function of the *SsSR* gene in *S. sclerotiorum*, *SsSR* deletion mutants (Δ*SsSR*-3 and Δ*SsSR*-4) were generated using a homology recombination strategy (Figure S1A). Complemented strains were generated by expressing an SsSR-flag fusion protein in the Δ*SsSR* background through polyethylene glycol (PEG)-mediated protoplast transformation [29]. The transformants obtained were verified by PCR (Figure S1B) and Western blot (Figure S1C). Comparison of the Δ*SsSR*-3 and Δ*SsSR*-4 mutants with the wildtype strain 1980 and complemented transformants (Δ*SsSR*-C-5 and Δ*SsSR*-C-6) revealed no significant differences in growth rate, oxalic acid production, or hyphal tip morphology (Figure 2A,B). These results suggest that *SsSR* is not essential for the growth or acid production of *S. sclerotiorum*.

To explore the role of *SsSR* in the pathogenesis of *S. sclerotiorum*, the aforementioned strains were inoculated onto leaves of *Brassica napus* and *Arabidopsis thaliana*. The Δ*SsSR*-3 and Δ*SsSR*-4 strains produced smaller lesions on *B. napus* compared with the wildtype strain 1980 and the *SsSR*-complemented strains, which caused severe lesions under identical conditions (Figure 2C,D). Relative biomass analysis further confirmed that the Δ*SsSR* strains had significantly reduced biomass compared with the wildtype strain 1980 (Figure 2E). Similar results were observed in the inoculation experiments using *A. thaliana* (Col-0), where the Δ*SsSR* mutants caused limited necrosis, in contrast to the extensive necrosis produced by the wildtype strain 1980 (Figure 2F,G). The relative biomass results were consistent with these observations (Figure 2H). These results indicate that *SsSR* is essential for the full virulence of *S. sclerotiorum*.

### 3.3. SsSR Promotes Infection by Regulating Ergosterol Synthesis

**Figure 3:**
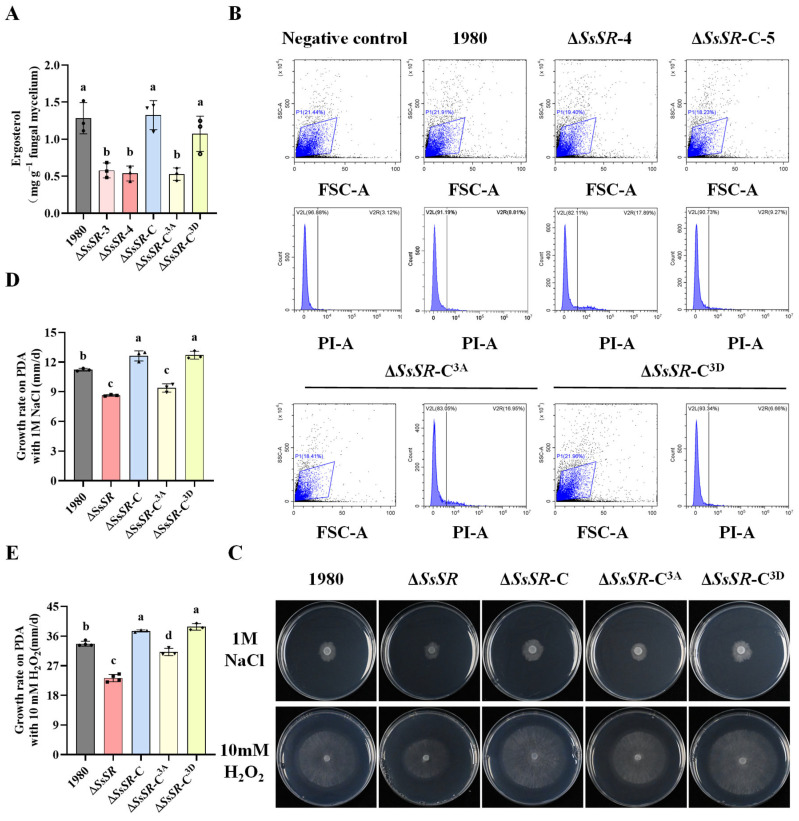
SsSR promotes infection by regulating ergosterol biosynthesis. (**A**) Relative content of ergosterol in the wildtype strain 1980 and in the Δ*SsSR*-3, Δ*SsSR*-4, Δ*SsSR*-C-5, and Δ*SsSR*-C-6 mutant strains after growth on PDA for 2 days at 20 °C. (**B**) Protoplasts of indicated strains were treated with a propidium iodide (PI) solution for 15 min, and cells with fluorescence were counted to represent cell membrane integrity. (**C**) Colony morphology of the wildtype strain 1980, *SsSR* mutants, and the complementary strains (Δ*SsSR*-C, Δ*SsSR*-C^3A^, and Δ*SsSR*-C^3D^) on PDA with 1 M NaCl and 10 mM H_2_O_2_ at 20 °C. (**D**,**E**) Growth rates of above strains on PDA with 1 M NaCl and 10 mM H_2_O_2_. Different lowercase letter (a, b, c, d) represented significant differences between groups (*p* < 0.05).

The deletion of FgSR, a homologous protein of SsSR in *F. graminearum*, causes a significant reduction in ergosterol synthesis [33]. To evaluate the role of *SsSR* in ergosterol biosynthesis, we obtained mycelia extracts from the wildtype stain 1980, *SsSR* mutants, and complemented strains for HPLC analysis. The results showed that Δ*SsSR*-3 and Δ*SsSR*-4 produced significantly less ergosterol compared with the wildtype and complemented strains, suggesting that *SsSR* plays a crucial role in ergosterol biosynthesis (Figure 3A). Given that ergosterol is a fundamental component of the cell membrane, we assessed plasma membrane integrity (PMI) in the Δ*SsSR*-3 and Δ*SsSR*-4 strains using propidium iodide (PI) staining of protoplasts followed by fluorescence-activated cell sorting. A higher proportion of cells in the Δ*SsSR*-3 and Δ*SsSR*-4 strains exhibited PI fluorescence compared with the wildtype strain 1980 and complemented strains (Figure 3B). These results demonstrated that *SsSR* is essential for ergosterol synthesis, which is vital for maintaining cell membrane integrity. Consequently, we tested the sensitivity of *SsSR* mutants to various stress conditions. Compared with the wildtype strain 1980 and complemented strains, the Δ*SsSR*-3 and Δ*SsSR*-4 strains exhibited significantly increased sensitivity to osmotic stress (1 M NaCl) and oxidative stress (10 mM H_2_O_2_) (Figure 3C–E). These results indicated that SsSR is necessary for maintaining cell membrane integrity and resistance to multiple stressors.

### 3.4. The Phosphorylation Sites of SsSR Were Indispensable for Virulence and Ergosterol Synthesis in Sclerotinia sclerotiorum

TFs often possess phosphorylation sites that necessitate activation by upstream kinases to fulfill their functional roles [37]. To investigate whether the transcription factor SsSR requires being phosphorylated during *S. sclerotiorum* infection, and to pinpoint the potential phosphorylated sites of *SsSR*, we utilized the NetPhos 3.1 Server (http://www.cbs.dtu.dk/services/NetPhos/, accessed on 15 May 2021) for analysis and identified three predicted amino acid sites that might undergo phosphorylation (Figure S2A). Subsequently, we generated two mutations in the *SsSR* gene and introduced them into the Δ*SsSR* strain, yielding two complementary strains: Δ*SsSR*-C^3A^, harboring a phospho-inactive SsSR (Ser/Thr phosphorylation sites replaced with alanine), and Δ*SsSR*-C^3D^, possessing a phosphomimetic SsSR (Ser/Thr phosphorylation sites replaced with aspartic acid) (Figure S2B).

**Figure 4:**
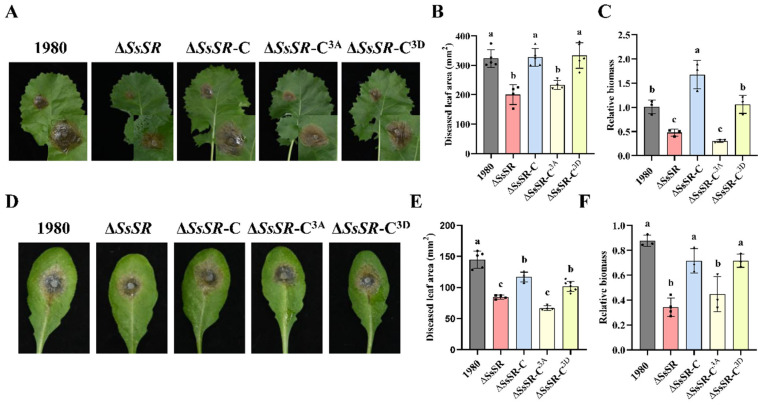
The phosphorylation sites of SsSR were indispensable for virulence and ergosterol biosynthesis in *Sclerotinia sclerotiorum.* (**A**,**D**) Disease symptoms on the detached *B. napus* and *Arabidopsis* leaves, respectively, inoculated with *Sclerotinia sclerotiorum* wildtype strain 1980, *SsSR* mutant strain, and complementary strains (Δ*SsSR*-C, Δ*SsSR*-C^3A^, and Δ*SsSR*-C^3D^). Photographs were taken at 36 h post-inoculation. (**B**,**E**) Lesion areas were measured by the cross-over method. (**C**,**F**) DNA was extracted from tissue samples of mycelium and leaves at lesion sites for detecting the number of pathogen and plant endogenous genes using RT-qPCR. Then, the CT values obtained were used to analyze relative biomass. Different lowercase letter (a, b, c) represented significant differences between groups (*p* < 0.05).

To determine whether the phosphorylation sites of *SsSR* are essential for virulence, the Δ*SsSR* strain and its different complemented strains (Δ*SsSR*-C, Δ*SsSR*-C^3A^, and Δ*SsSR*-C^3D^) were inoculated onto plant leaves. Both the Δ*SsSR* strain and the nonphosphorylatable complementary strain Δ*SsSR*-C^3A^ exhibited significantly reduced virulence on *B. napus*, producing smaller disease lesions compared with the wildtype strain 1980 and the complementary transformants (Δ*SsSR*-C and Δ*SsSR*-C^3D^), which caused serious necrotic lesions (Figure 4A,B). The relative biomass analysis of the lesion area showed consistent trends (Figure 4C). Similarly, the virulence of the Δ*SsSR* strain and the nonphosphorylatable complementary strain Δ*SsSR*-C^3A^ were also decreased on *A. thaliana* (Col-0). The lesion area of the phosphomimetic complementary strain Δ*SsSR*-C^3D^ was comparable to that of the wildtype strain 1980 (Figure 4D–F). These results indicate that the phosphorylation sites of SsSR are indispensable for virulence in *S. sclerotiorum*.

To further investigate the role of these phosphorylation sites of SsSR, we analyzed the relative ergosterol content, cell membrane integrity, and stress resistance in the phosphomimetic and nonphosphorylatable complementary transformants (Δ*SsSR*-C^3D^ and Δ*SsSR*-C^3A^). Compared with the wildtype strain 1980, the nonphosphorylatable complementary strain Δ*SsSR*-C^3A^ exhibited significantly reduced ergosterol content, compromised membrane integrity, and decreased stress tolerance. In contrast, the phosphomimetic strain Δ*SsSR*-C^3D^ showed no noticeable differences from the wildtype strain 1980 (Figure 2A–E). These results suggest that the phosphorylation sites of SsSR are also crucial for stress tolerance in *S. sclerotiorum*.

### 3.5. SsSR Regulates the Transcription of Genes Involved in Sterol Synthesis During Infection

**Figure 5:**
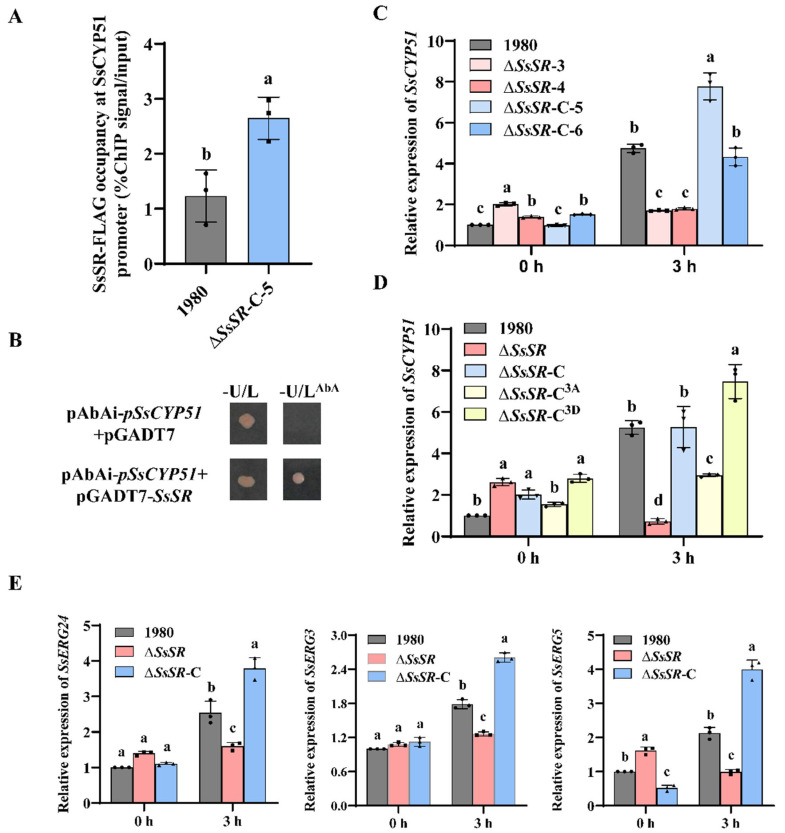
SsSR regulates the transcription of multiple genes involved in sterol biosynthesis during infection. (**A**) ChIP assay was used to indicate f SsSR binds to the SsCYP51 promoter in the complementary strain (Δ*SsSR*-C-5). A control reaction was processed in parallel with the wildtype strain 1980 as a negative control. (**B**) Yeast one-hybrid (Y1H) assay was used to indicate SsSR binds to the *SsCYP51* promoter. The *SsCYP51* promoter was used as the bait and the pGADT7-SsSR as the prey. (**C**) Relative levels of transcript accumulation of *SsCYP51* in the wildtype strain 1980, Δ*SsSR* strains (Δ*SsSR*-3 and Δ*SsSR*-4), and complementary strains (Δ*SsSR*-C-5 and Δ*SsSR*-C-6) were determined by RT-qPCR when inoculated on rapeseed leaves. The expression level of *SsCYP51* in the wildtype strain 1980 after inoculation of leaves for 0 h was normalized to 1. (**D**) Relative levels of transcript accumulation of *SsCYP51* in the wildtype strain 1980, Δ*SsSR* strain, and complementary strains (Δ*SsSR*-C, Δ*SsSR*-C^3A^, and Δ*SsSR*-C^3D^) were determined by RT-qPCR when inoculated on rapeseed leaves. The expression level of *SsCYP51* in the wildtype strain 1980 after inoculation of leaves for 0 h was normalized to 1. (**E**) Relative levels of transcript accumulation of ergosterol-related genes (*SsERG24*, *SsERG3,* and *SsERG5*) in the wildtype strain 1980, the complementary stain (Δ*SsSR*-C), and the Δ*SsSR* strain (Δ*SsSR*-3) were determined by RT-qPCR when inoculated on rapeseed leaves. The expression level of each gene in the wildtype strain 1980 after inoculation of leaves for 0 h was normalized to 1. Different lowercase letter (a, b, c, d) represented significant differences between groups (*p* < 0.05).

A previous study reported that FgSR, a homologous protein of SsSR in *F. graminearum*, enhances resistance to azole fungicides by regulating the expression of *FgCYP51* to promote ergosterol synthesis [33]. The 14-α-demethylase *CYP51* gene encodes a key enzyme in sterol biosynthesis [38]. To investigate whether *SsCYP51* has a conserved function and is regulated by SsSR, we conducted chromatin immunoprecipitation–quantitative PCR (ChIP-qPCR) assays using an SsSR-Flag-tagged strain (Δ*SsSR*-C-5). The results showed that SsSR-Flag was significantly enriched at the *SsCYP51* promoter, with no enrichment observed in the negative control (Figure 5A). Furthermore, yeast one-hybrid (Y1H) assays were performed to evaluate the binding ability of SsSR to promoters of *SsCYP51*. Results showed that co-expression of SsSR with the *SsCYP51* promoter moderately activated the reporter gene expression (Figure 5B). Collectively, these results suggest that the transcription factor SsSR binds to the promoter of *SsCYP51* to mediate its transcription.

In view of the reduced virulence and ergosterol content in the Δ*SsSR* strains, we speculated that Δ*SsSR* strains may fail to effectively induce *SsCYP51* expression during infection. As expected, RT-qPCR assays showed that the transcription of *SsCYP51* was significantly induced in the wildtype strain 1980, the complementary strains (Δ*SsSR*-C-5 and Δ*SsSR*-C-6), and the phosphomimetic complementary strain Δ*SsSR*-C^3D^, but not in the ΔSsSR strains (Δ*SsSR*-3 and Δ*SsSR*-4) or the nonphosphorylatable complementary strain ΔSsSR-C^3A^ after plant inoculation (Figure 5C,D). This indicates that phosphorylated SsSR regulates *SsCYP51* expression during infection. In addition, other ergosterol biosynthesis genes (*SsERG3*, *SsERG24,* and *SsERG5*) were also upregulated during infection in the wildtype strain 1980 and the complementary strains, but not in the Δ*SsSR* strain (Figure 5E). Taken together, these results confirm that SsSR regulates the transcription of genes involved in sterol synthesis, including *SsCYP51*, during infection.

### 3.6. Regulation of SsCYP51 by SsSR Is Essential for Virulence and Ergosterol Biosynthesis in Sclerotinia sclerotiorum

**Figure 6:**
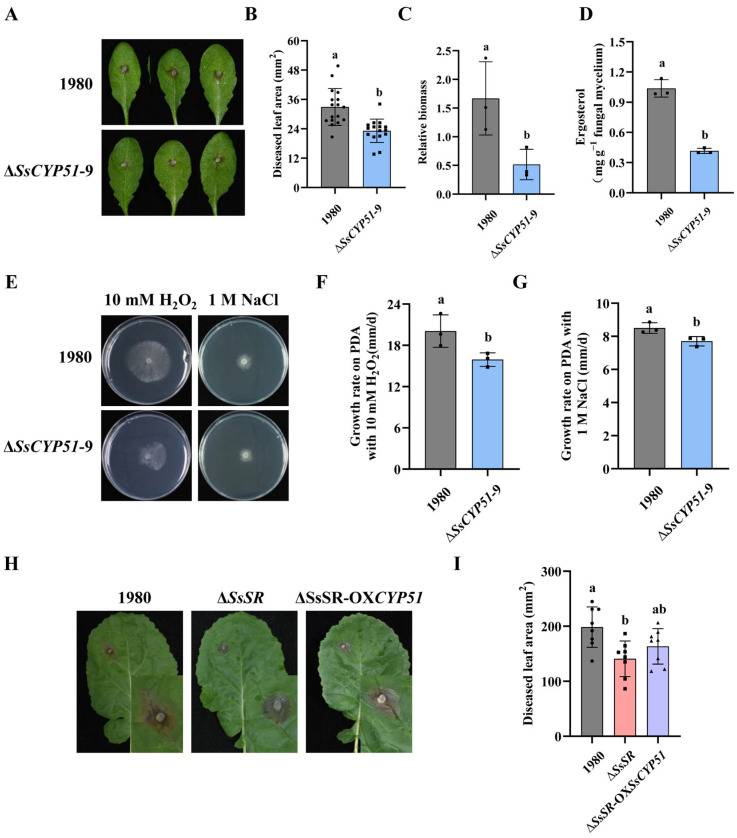
Regulation of SsCYP51 by SsSR is essential for virulence and ergosterol biosynthesis in *Sclerotinia sclerotiorum.* (**A**) Disease symptoms on the detached *Arabidopsis* leaves inoculated with *Sclerotinia sclerotiorum* wildtype strain 1980 and *SsCYP51* mutant strain. Photographs were taken at 24 h post-inoculation. (**B**) Lesion areas were calculated by the cross-over method. (**C**) DNA was extracted from samples of mycelium and leaves at lesion sites to detect the number of pathogen and plant endogenous genes using RT-qPCR. Then, the CT values obtained were used to analyze relative biomass. (**D**) Relative content of ergosterol in the wildtype strain 1980 and *SsCYP51* deletion strain after growth on the PDA for 2 days. (**E**) Colony morphology of the wildtype strain 1980 and *SsCYP51* mutant strain on PDA with 1 M NaCl and 10 mM H_2_O_2_ at 20 °C. (**F**,**G**) Growth rates of above strains on PDA with 1 M NaCl and 10 mM H_2_O_2_. (**H**) Disease symptoms on the detached *Arabidopsis* leaves inoculated with *Sclerotinia sclerotiorum* wildtype strain 1980, *SsSR* mutant strain, and Δ*SsSR*-OX*SsCYP51*. Photographs were taken at 36 h post-inoculation. (**I**) Lesion areas were calculated by the cross-over method. Different lowercase letter (a, b) represented significant differences between groups (*p* < 0.05).

To elucidate the function of *SsCYP51*, we generated an *SsCYP51* mutant strain, and it was verified by PCR (Figure S3A). When the Δ*SsCYP51* strain was inoculated onto Col-0 to assess virulence, we observed smaller lesions compared with those caused by the wildtype strain 1980 (Figure 6A,B). The relative biomass measurements were consistent with the lesion areas observations (Figure 6C). Moreover, as a gene regulated by SsSR, the ergosterol content in the *SsCYP51* mutant strain was also significantly lower than in the wildtype strain 1980 (Figure 6D). These data indicate that *SsCYP51* is involved in virulence and ergosterol synthesis. In addition, we evaluated the impact of *SsCYP51* on various phenotypes. When cultured on PDA, mycelial tip morphology, oxalic acid production, sclerotial formation, and hyphae growth of the wildtype strain 1980 and Δ*SsCYP51* strain were observed. Notably, the *SsCYP51* mutant exhibited no apparent difference in these traits compared with the wildtype strain 1980 (Figure S3B,C), suggesting that *SsCYP51* is not crucial for growth, acid production, mycelial tip morphology, or sclerotium formation. However, the absence of *SsCYP51* resulted in a substantial increase in sensitivity to oxidative stress and osmotic stress compared with the wildtype strain 1980 (Figure 6E,G). These results indicate a role for *SsCYP51* in stress tolerance.

To further validate the function of *SsCYP51* as a gene regulated by SsSR, we overexpressed *SsCYP51* in the background of the *SsSR* mutant strain, which was confirmed by Western blot analysis (Figure S4A). We found that Δ*SsSR*-OX*SsCYP51* has comparable growth rates under osmotic and oxidative stress conditions to the wildtype strain 1980, suggesting that overexpression of *SsCYP51* can compensate for the defects in stress resistance observed in the Δ*SsSR* strain (Figure S4B,C). We also assessed the virulence of the wildtype strain 1980, the Δ*SsSR* strain, and Δ*SsSR*-OX*SsCYP51* on Col-0. Results showed that the Δ*SsSR*-OX*SsCYP51* mutant partially restored virulence (Figure 6H,I). These results further suggested that SsSR mainly affects the virulence and stress tolerance of *S. sclerotiorum* by regulating the expression of *SsCYP51*. Of course, SsSR may also regulate other genes related to virulence, as the virulence of Δ*SsSR*-OX*SsCYP51* did not completely recover.

## 4. Discussion

TFs regulate gene expression by binding to DNA motifs to sense the external environment and control fungal growth and virulence, linking the perception of external signals to transcriptional reprogramming [20,39]. In this study, we identified a transcription factor, SsSR, whose deletion in mutants does not affect growth but significantly reduces virulence (Figure 2). The homology analysis revealed that SsSR is very closely related to the transcription factor FgSR, which also contains a zinc finger domain and a fungal-specific transcription factor domain. Deletion of *FgSR* results in a significant reduction in ergosterol in *F. graminearum* [33]. To investigate whether SsSR has a similar regulatory function on ergosterol synthesis, the ergosterol content of each strain was determined. The HPLC results also showed a significant reduction in ergosterol levels in the *SsSR* knockout strains (Figure 3A), suggesting that SsSR functions to regulate ergosterol synthesis.

Previous studies have found that inhibition of ergosterol synthesis tends to affect the growth and virulence of pathogens. In *Aspergillus fumigatus*, *M. oryzae*, and *F. graminearum*, deletion of genes involved in ergosterol synthesis leads to a decrease in virulence, which mainly results from reduced growth [40,41,42]. However, it has also been found that deletion of ergosterol-regulated transcription factor *BcSR* does not affect growth in *B. cinerea* [33]. In our study, Δ*SsSR* strains did not affect growth (Figure 2A,B). This suggests that, unlike in some pathogens, the reduction in virulence of Δ*SsSR* strains is not due to a growth defect caused by ergosterol deficiency. As a major component of the cell membrane, impaired ergosterol synthesis can also lead to programmed cell death (apoptosis and macrophage) or reduced stress resistance [43,44]. *SsSR* deficiency appears to contribute to compromised phenotypes related to ergosterol-dependent traits, including reduced cell membrane integrity and decreased stress tolerance (oxidative and osmotic stress) (Figure 3B–E). Plant–pathogen interactions lead to changes in the plant’s microenvironment, such as hyperosmolarity and ROS production [45]. Therefore, reduced resistance to stress may also be associated with reduced virulence. Other studies have also identified ergosterol’s role in stress resistance. For example, the loss of *ERG6P* reduces ergosterol synthesis and increases sensitivity to oxidative stress and high iron environments [46]. Consequently, SsSR controls virulence in *S. sclerotiorum* by regulating ergosterol biosynthesis and response to environmental stresses.

To investigate which genes are transcriptionally regulated by SsSR during infection, we have demonstrated the binding of SsSR to the promoter of *SsCYP51*, a gene related to ergosterol biosynthesis, by ChIP-qPCR and Y1H assays (Figure 5A,B). Furthermore, SsSR can regulate the expression of *SsCYP51* during the infection (Figure 5C,D). Importantly, we found that the phenotypes of *SsCYP51* knockout mutants were similar to those of *SsSR* mutants, with lower ergosterol content and reduced virulence (Figure 6A–F), suggesting that SsSR regulates *SsCYP51*, thereby influencing ergosterol synthesis and virulence in *S. sclerotiorum*. To further define the function of *SsCYP51*, by overexpressing *SsCYP51* in the background of the *SsSR* mutant strain, the resulting transformant, Δ*SsSR*-OX*SsCYP51*, fully restored stress resistance but only partially restored virulence (Figure 6H,I and Figure S4). Therefore, we confirm that the transcription of *SsCYP51* is indeed regulated by SsSR. However, it is plausible that SsSR may also regulate the expression of other genes involved in virulence that remain undiscovered.

The transcription of genes related to ergosterol synthesis (*SsCYP51*, *SsERG3*, *SsERG5,* and *SsERG24*) is regulated by SsSR (Figure 5C–E). Gene cluster co-regulation is a well-established mechanism especially prominent in metabolic pathways like antibiotic, toxin, and secondary metabolite synthesis [47,48]. In fungi, transcription factors that regulate the biosynthesis of secondary metabolites are often frequently clustered together with the biosynthesis-related genes [49]. Upon examining the genomic locations of *SsSR*, *SsCYP51*, *SsERG3*, *SsERG5*, and *SsERG24*, we found that most of these genes are not positioned on the same chromosome. Although the genes *SsERG5* and *SsSR* are both located on chromosome 1, they are situated quite far from each other. This observation suggests that the TF SsSR and the enzymes it regulates do not follow the gene clustering pattern. During the infection process of *S. sclerotiorum*, SsSR has the ability to activate genes encoding enzymes involved in the ergosterol biosynthesis pathway even though these genes are scattered at different locations across the genome.

We found that SsSR possesses three phosphorylation sites (Figure S2A). The activity of TFs is commonly regulated by upstream kinases, with the MAPK cascade pathway being a notable example that regulates transcriptional activity by modulating the phosphorylation of TFs [37]. In our study, the transcriptional activity of SsSR was indeed regulated by phosphorylation, and its phosphorylation sites were essential for virulence in *S. sclerotiorum*. Specifically, the phosphomimetic complementation transformant Δ*SsSR*-C^3D^ exhibited virulence comparable to the wildtype strain 1980, whereas the nonphosphorylatable *SsSR* complementation strain Δ*SsSR*-C^3A^ showed significantly reduced virulence (Figure 4). This suggests that the phosphorylation site on the SsSR is critical for virulence. Analogously, in *F. graminearum*, the transcription factor FgSR was also phosphorylated to enhance the transcription of genes involved in sterol synthesis. When FgSR could not be phosphorylated, its ability to resist azoles and the transcription levels of *FgCYP51* was markedly decreased [33]. In this study, we found that the expression level of *SsCYP51* in Δ*SsSR*-C^3A^ was significantly lower than that of the wildtype strain 1980 and Δ*SsSR*-C^3D^ during infection (Figure 5D). This further confirms the role of *SsSR* and *SsCYP51* in virulence. To further elucidate the function of the phosphorylation site on *SsSR*, we conducted additional experiments. Results showed that the ergosterol relative content, cell membrane integrity, and stress resistance of Δ*SsSR*-C^3A^ were comparable to those of knockout transformants (Δ*SsSR*-3 and Δ*SsSR*-4), which were substantially reduced compared with the wildtype strain 1980 and Δ*SsSR*-C^3D^ (Figure 3A–E). These results genetically confirm the function of SsSR and its phosphorylation sites, reinforcing the importance of phosphorylation in regulating SsSR’s activity.

**Figure 7:**
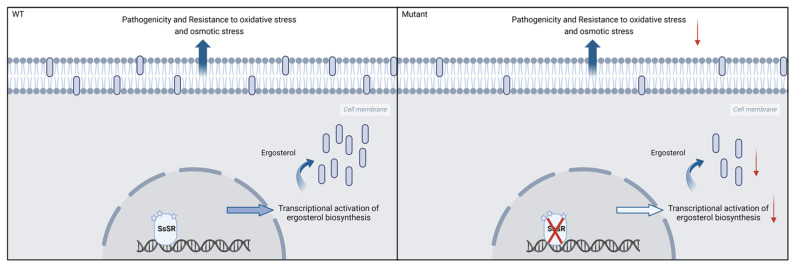
A proposed model depicting the function of SsSR in virulence, tolerance to stress, and ergosterol synthesis. During the infection process, SsSR was phosphorylated to bind the promoters of ergosterol biosynthesis-related genes, inducing the high transcription levels of sterol biosynthesis genes and promoting ergosterol synthesis. However, in the *SsSR* mutant strains, deletion of SsSR resulted in significantly lower expression levels of ergosterol synthesis-related genes than in the wildtype strain 1980, and ergosterol synthesis was markedly reduced, affecting cell membrane integrity, which reduced the pathogenicity and tolerance to stress of *S. sclerotium*. The red arrows represent reduced level. Figure created using BioRender (http://biorender.com/, accessed on 15 May 2021).

In conclusion, we have identified a zinc finger transcription factor, SsSR, that is essential for virulence but not for growth in *S. sclerotiorum*. The phosphorylation status of *SsSR* regulates its transcriptional activity. Specifically, phosphomimetic SsSR enhances ergosterol synthesis by inducing the transcription of *SsCYP51* and other genes involved in ergosterol synthesis, thereby facilitating infection and stress resistance. Conversely, the absence of SsSR results in decreased ergosterol synthesis and compromised cell membrane integrity, ultimately impairing virulence and stress tolerance (Figure 7). Our findings highlight the crucial role of *SsSR* in the virulence of *S. sclerotiorum* and offer a theoretical foundation for the development of potential drug targets.

## Data Availability

The authors confirm that the data supporting the finding of this study are available in the article, and the original data presented in the study are openly available in FigShare at https://doi.org/10.6084/m9.figshare.28749989.

## Supplementary Materials

The following supporting information can be downloaded at: https://www.mdpi.com/article/10.3390/jof11070509/s1, Figure S1: Construction and verification of the Δ*SsSR* mutants (Δ*SsSR*-3 and Δ*SsSR*-4) and the complementary mutants (Δ*SsSR*-C-5 and Δ*SsSR*-C-6); Figure S2: Generation and confirmation of the dephosphorylation complementary strain Δ*SsSR*-C^3A^ and the phosphorylation complementary strain Δ*SsSR*-C^3D^; Figure S3: *SsCYP51* is not necessary for mycelial growth in *S. sclerotiorum*; Figure S4: Overexpression of *SsCYP51* in the *SsSR* mutant background restored stress resistance; Table S1: PCR primers used in this study; Table S2: RT-qPCR primers used in this study.
