# Supplementary material for: The Transcription Factor SsSR Mediates Ergosterol Biosynthesis and Virulence in Sclerotinia sclerotiorum
Source: J Fungi (Basel). 2025 Jul 5;11(7):509. doi: 10.3390/jof11070509 (PMC12299684; doi:10.3390/jof11070509)
Supplement: Supplementary file 1 [file jof-11-00509-s001.zip › S3_data.pdf]

SsSR:
atgccaccaagaagatcgcataagaaatccaaagccggatgccaaagatgcaaacataggaagatcaagtgtgatgaaa
aacatcccttgtgtggaaattgccaaaaacatggcgtttcctgtgactttgccgacaacccatcgtcacctgactcaccagcc
ttccagcctccatcgctatctcaccaaacttcagatgaatctgtggcccatgtatcatcaccagcttctaccaattcgtcgataa
tgccattctatcggtcccctgcacagatgttcaggggccatagtaatacggtacatgatcgatcacttgagctcaagcttatgc
accattatacagcagcgacttcgaaaactctatctaatgatgaaagtcaggaagttgcttggtctgtcaacgtaccgtcgctc
gcttataattctccattttgatggatgctattcttgctgtttctgctctacatttacgtatattacaacccgatgatcgaagtctcgta
cgtgcttcacatagttacatggcatcggcgcttgctcaatattcttccctgttgaacagaggtccatccgaaagcaatgcaga
agctctttttgtacagctgctctcattgcttttcaagcatcggcatcccgctgtgtcgagaatggaattgacggtgattctgata
gtggttatgtctttccactcgcttggtttcattcttttcaaggggtgaagacagtcgtcataacttcctggcaatggttacggaatt
ccgagaaaatatttcccatcatcaatgggcagcctccattaatactggatcttgatcctgagcgccgagctttctttgcttattta
ctcgaaggtatcgacaacgaatcaaggtcaaatgatgcttacataaatgcggatacgaagcgggcatacgaacatgccgtt
gcgatgctaaactgggcccaccaaaaaccagagagagctcgaatccttggctttgcggccaccgtatcgcgccgttatatt
gagctgttaaaggaacaggatcctcgtgctttagtcatttctgcatgtttctttgctatgacacgcgtcgttgatgaaatttggtg
gcttcaaggtgttgccaagaaggaagtggctggaattttcagtttgttaccggaagagtggtggcctaagatggattggccg
atgaaaatttcacattttgagggaaagctagatgagaatacttggggcacttgttggcattctgacggaacgccaaaacgag
aagaaggcttcaatggcaatcttatctcgcatattgacatgttggcacaaattttgaaccaaaccgcaccgccaccagattga
SsCYP51:
atgggtattctcgaaacaattgccgggccattggctcaagagatttcgcaaaggtcaacctttgctgttgttgctgctggcgtg
gcagcattcgtcgttctatctgtcattctcaatgtcctgaatcaagtgttatttgcgaaccccaatgaaccaccagtggtcttcca
ctggtttccaatcattggtagcaccgtcacttatggtatggacccttataaattcttcttcgagtgtcgcgcaaaggtacgctttc
catccccgtgttcattgcttggagactaatgtttgtgtattagtacggtgatattttcacatttgtcttgctcggaaagaagaatac
agtatatcttggacgaaatggcaatgactttattctcaatggcaagcttaaggatctcaatgcggaggaaatatatactgttttg
acaactcccgtgtttggaaaggatgtagtctacgattgccccaatgcgaaattgatggagcaaaaaaaggtgggttgttaata
ggcgatagaaagacgagttgatggctaattgtgctgcaatagttcatgaaaattggcttgtctactgaagctttccgatcctac
gtcccaattatacaaatggaagtggaaaacttcatgaaacgttcttcggtattcaagggacaaaagggaactgccgatattg
gtcccgctatggctgaaatcaccatctataccgcttcgcatactctacaaggaaaggaagtccgtgatcgatttgatactactt
tcgcctctctctaccacgaccttgatatgggctttagtcccatcaactttatgcttcactgggctcctcttcctcacaaccgtgcc
cgcgaccatgcgcagagaactgtcgcagcaacatatatggatattattaaaaaacgacgtgctcaggctacggaagccga
cttcaaatccgacattatgtggcaattgatgcgctcgtcctacaaagatggaaccccccgttccagaccgagagattgctcac
atgatgatcgctcttctcatggccggacagcactcttcctcatcttctatctcttggattctgcttcgtcttgcctcacgcccagat
atcatggaagaactctatcaagaacaaatccaagttctgggcgccgatctccctgctctcaagtacgaggacctggccaaa
cttcctcttcatcaaaacatcttgaaggaaactctccgcatccacactcccatccattctattatgcgcaaagtcacaacacca
atgccaattagcggaacaaaatatgtcattccaacctcgcatactcttatggcatctcctggttgtacaagtcgagacgcggat
tacttcccagagccacttgagtgggaccctcatagatgggacattggctcgggccgtgtaattggcaatgatcaggacgaa
gaattccaagattatggctatggaatgatcagcaaaggtgcttctagtccttaccttccattcggtgctggcagacacaggtgt
atcggtgaacaattcgccaatgtacagctcatcactatcatggccactgtggttagaatgttcaaattcaagaacgttgatggc
agcaaggatgtcattggtactgattacaccagtttattcaccaggccattggcgccagcagttatagcatgggagcgacgat
aa
SsERG3:
atggatatcgctttggaacttaccgataaatacttcttcgatcatttctattcagccttactacctgcgaaatctccattgttaaatg
cgattggggaacaaattaacgggacaactctcaatgcgaaaacagcttcaacatggcaatatcatccttcgaattctttttgc
aattcgaacctacccatgctgcatacacaagtcaatgggatagagataatatctatcgacaagctttctcattgttcttgattact
tggtatgtttattactttcgacgggcatccaagagatgtatattacatatcaaatactgattgttaaataggatcgcaggtgcagc

tctctactttacaatcgcgactctatcctacatcttcgttttcgacaaaactacctttcaacatcctaaattcctcaaaaatcaaatt
cgcatggaaatctcccaggcttgcaatagtatgccattgatgtctatcctcactacacctttcttcctcgcagaagtaagaggtt
actccaaactttacgaccttacatctgaaggacctggttcatggtataattggtttcaattcccactcttcctagccttcaccgatt
gcttcgtctacttcatccatcgaggtctacaccatccttcggtctacaaaactctccataagccacatcataaatggattatgcc
aactccatacgcctccattgctttccaccccgttgatggttggatgcaatcacttccttaccacgtcttccccttcatcttcccact
tcagaaattcgcctacctgggtcttttcttctttgttcagatctggacggtttttatccatgatggtgaatatgtagccaattctcct
attcttaatggagcagcttgtcatactatgcatcatctttactttaactataattatggtcaatatacgacattgtgggatcgtctag
gaggtagttacagaaaacctaacgaggaacttttcagaagggaaagtaagatgggtcaaaaggaatgggagagacaagtt
gcggaaatggaaaaacaagttttagaggctgagggagtagatgatagagtttatgatgttggggagaagaagaagaattag
SsERG5:
atggcggaccttgtacaaaacatcacttatgcctcacctgtggcagatgcaaaactggctcaagccattgggaatcctcagc
tcaatagctatctggccaaactcaccgaagtcagcgcatggcagattgtccttacaattctttgtctgatggtagcttatgatca
atgtacaaatccccggcgccaagaactcactcactcgcgcgatcactcaatcaatcaattgcatcactgacatctctgcaggt
agttatatttggcaaaaaggttcaattatcggtccagcatggaaagccccctttatcggtccatttctacaatctgtcaatccaa
aattccacgaatatcaagcaaaatgggcaagcggcgatctcagttgtgtatcggttttccacaagtaagtttataccatcatcc
gccactcgatatttccaggctctgaccattttcagattcgttgttattgcttctactcgtgatatggctagaaaggttttcaattctc
catcatacgttaagccatgtgtagtcgattcagcacacaagttgttgggtgagaccaactgggttttccttgatggaaaggcc
cacgttgatttccgaaagggtctcaatggattgtttacccgcaaagccctcgactgctacctcccaggacaagacgacgttta
caatcaatatcaagaacgattcgccaaggttaccgaggaagccggtggaaaacccgttccattcatgtccgaattccgtgaa
ttattgtgtgctgtttcctgccgtacattcgttggtttttacatctcagacgaagctatcaagaagatcgccgatgattactacaat
attaccgctgctttggaattggtcaatttcccaatcattcttcccttcaccaaatcctggtatggaaaaatcgctgccgacatggt
cctcgaggaattttccaagtgtgctgcaaagagcaaggttcgcatggctgccggtggtgaagtgtcttgcattatggatggat
gggtcaagtctcagcttgagtcggccgcttggcgcgaggcggaagccaagggtctttcaacagaagggatgacaaaacc
aacaccattacttcgtgactttaccgactacgaaatcgctcaaaccgtcttcaccttcttgtttgcttctcaagatgctactagca
gtgccgccacctggttattccaagttatggctcaacgtcctgaaattttagacaaggtccgcgaggaaaacttacgtgttcgt
ggtggtgataaacgagttcgaccaaacatggatatgatggaaagcatgaaatatactcgtgctgttgttagagaattgttgag
atggcgccctcctgttcttatggttccatatgtcgccaagaaggcattccctattactccagattacactgtccctaaaggttgg
tcaagctaacatgaaattatgatgcgatgctaatgagttaaataggagctatgatcgttccaacaacatatccagctttacgcg
accctgatgtctatgaccgacctgatgaatttgaccccgaacgttatttctctggtgatgccgaagtaaagggagctaagaac
tacttggtctttggaactggtgctcattattgtctgggccaagaatacgcacaaatgaaccttgctcttatgattggaaaggctt
caatggaacttgattgggttcatcatccaactcctaaatcagaagagatcaaagtctttgctacaatcttccctatggttagtac
atattcaaatggaaatcaattttcgagcatcactaactcaaattacaggatgattgtccattgaccttcacaaagagaccttaa
SsERG24:
atgactccaaagaagactaccacagtctctaaagcggctgagcctcatggctatgaatttggtggaccgtaagtcgcacaat
cccttgcagattcactgatctgtactgattatcgcagaataggagcttttggtatatccttcggacttccccttctctgctacgcg
acgacgtttctctgcaatgatatttctggatgtccagttccctcctcgctctccccttcgacccttacaatcaacagtctaaaaca
agaggttggatggccagaaaatggacttgcgggtttgtcaagttgggatgtatttctcaaagtagtaggatactactttctaag
catggtcttgcataggatcttgccgggcgaggaaaagctaggtgttgagcttgctagtgggggaaagctcaaatacaagttc
aatagtacgtacatctgctatacacgcgccattggtcgggacgctattcgctaatgcataagatagcttggtcttctacgatgtt
tacattcgctctatgcgccgcaggaactatcgcccagggagcagatttcccagtctggacttttatctacgacaattacctaca
aatccttacagccaatatcatcatctcctacggtcttgccacttatgtatatatccgcagttttagcatcaaacctggcaatcccg
agctccgcgaattggctgctggaggtcataccggaaatatgctatacgattggtttattggacgtgaactcaacccaagagta
actctccctttacttggcgagattgatatcaaggaattttgtgagctacgaccaggtcttatgggatggcttttaatggattacgc
atttgttgcccatcaatacaagacttacggatacgttaccgatagtatcttgctaattacagccttccaaacactgtatgtgcttg

attcatattggatggaaaatgccatcctcaccaccatggatattaccactgatgggtttggactcatgctatcatttggagactt
ggtctgggttccattcatctattctctccaagcacgttacctagccgtttacccccttctcttggcgttcttggcaccagcggtg
tcctcgctgttctaggcatcggatattacatcttccgtagttccaacaacgaaaagaaccgcttcagaaccgacccgacagat
gcccgtgtcgcacaccttaaatacctagagacgaaatccggctccaaacttctcatttcgggctggtggggtgtagctcgtc
atatcaactatgctggtgattggattatggcttggtcttactgtcttcccaccggtatcgctggctatctcatcaatcatagctcg
gtagctcccacatcaggccaatctctcgtcgacggttcatttgtgtatagtggaccagcagtgcacacggaagtcacacaag
gggcagctagaggctggggaatgatcattacatatttctatgtggtttactttggtgtcttgttggtgcatagagaaatgaggg
atgaggagaaatgcgaaagaaagtacggtgaggattggaagaggtataaggagattgtcaggtacagaattatcccggga
atctattaa
