# Supplementary figures and images for: The Transcription Factor SsSR Mediates Ergosterol Biosynthesis and Virulence in Sclerotinia sclerotiorum
Source: J Fungi (Basel). 2025 Jul 5;11(7):509. doi: 10.3390/jof11070509 (PMC12299684; doi:10.3390/jof11070509)

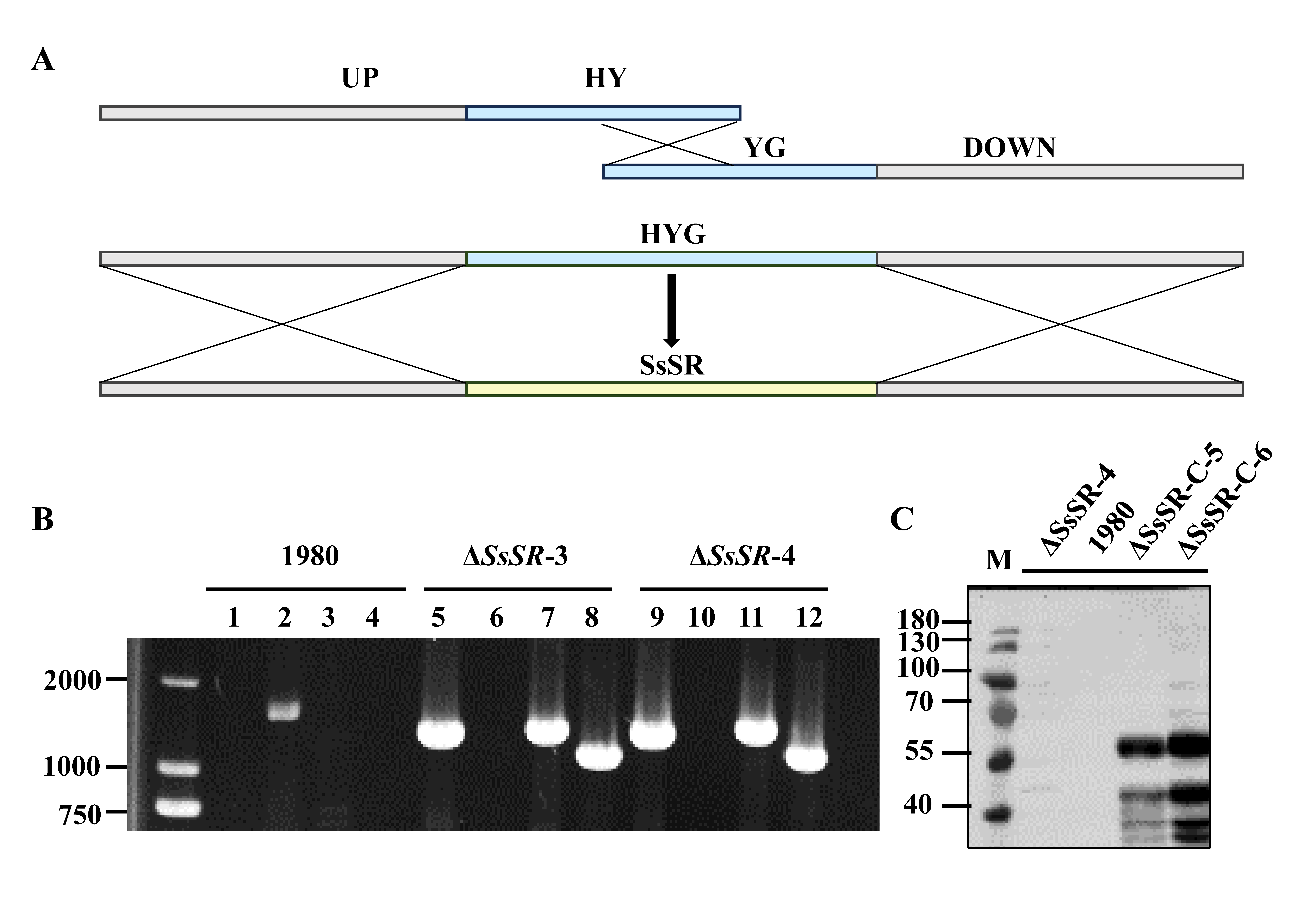

Supplement: Supplementary file 1 [file jof-11-00509-s001.zip › FIG.S1.tif]

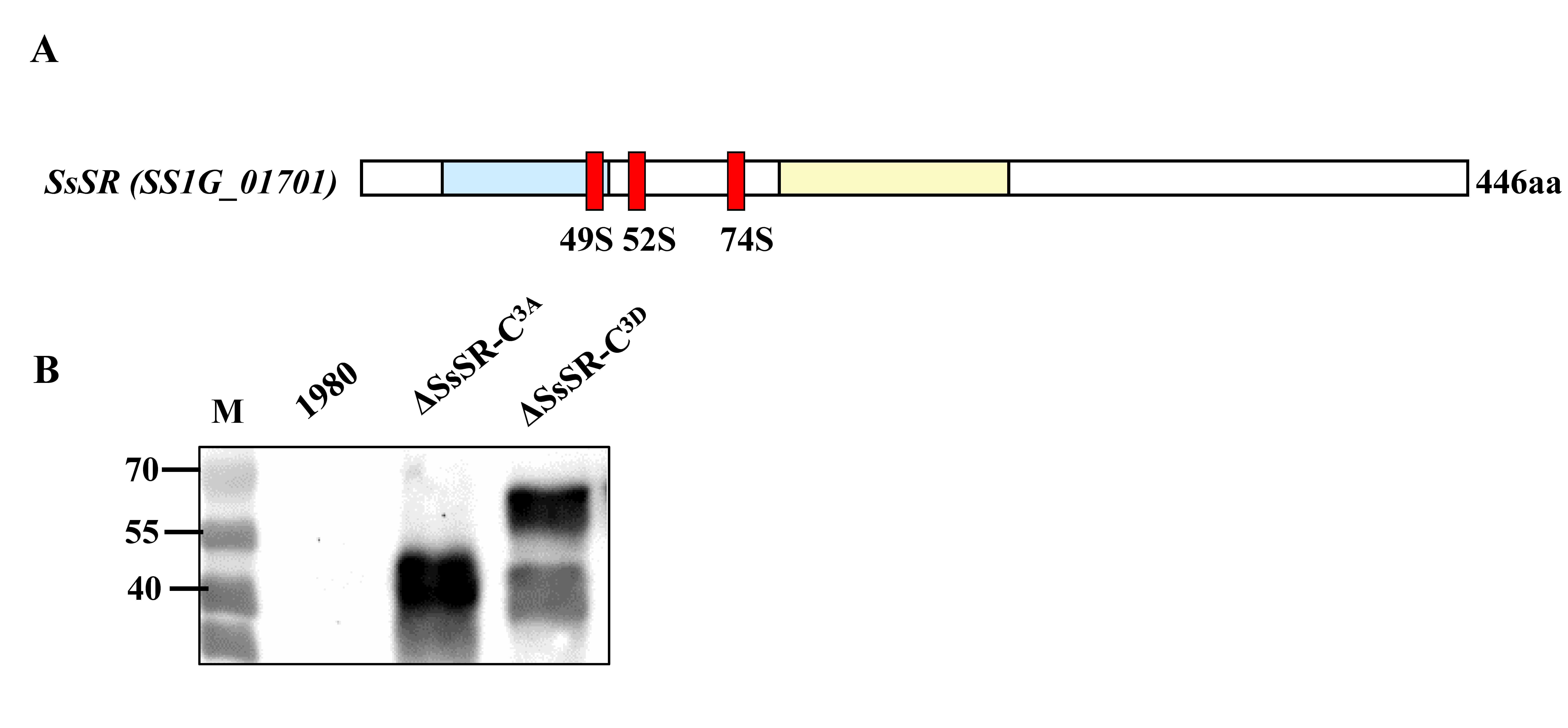

Supplement: Supplementary file 1 [file jof-11-00509-s001.zip › FIG.S2.tif]

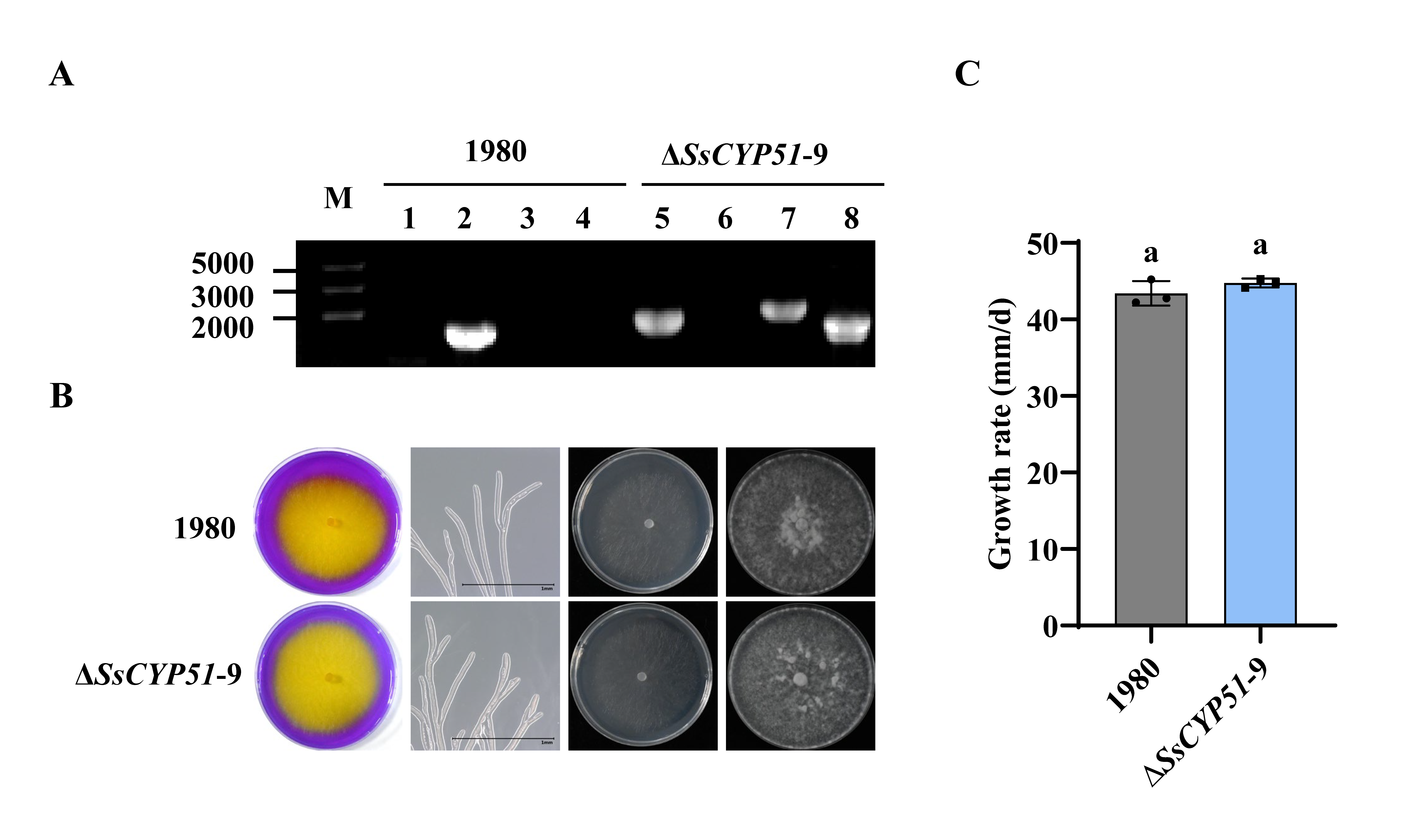

Supplement: Supplementary file 1 [file jof-11-00509-s001.zip › FIG.S3.tif]
